# An evaluation of emerging vaccines for childhood meningococcal disease

**DOI:** 10.1186/1471-2458-11-S3-S29

**Published:** 2011-04-13

**Authors:** Debajeet Choudhuri, Tanvir Huda, Evropi Theodoratou, Harish Nair, Lina Zgaga, Rachel Falconer, Ivana Luksic, Hope L Johnson, Jian Shayne F Zhang, Shams El Arifeen, Christopher B Nelson, Ray Borrow, Harry Campbell, Igor Rudan

**Affiliations:** 1 Centre for Population Health Sciences, Global Health Academy, The University of Edinburgh, UK; 2 International Centre for Diarrhoeal Disease Research, Bangladesh, Dhaka, Bangladesh; 3 Public Health Foundation of India, New Delhi, India; 4 Department of Clinical Microbiology and Hospital Infections, University Hospital Dubrava, Croatia; 5 Department of International Health, Johns Hopkins Bloomberg School of Public Health, Baltimore, MD 21205, USA; 6 Department of Immunizations, Vaccines and Biologicals, World Health Organization, Geneva, Switzerland; 7 Vaccine Evaluation Unit, Health Protection Agency, Clinical Sciences Building, Manchester Royal Infirmary, Manchester, M13 9WZ, UK; 8 Croatian Centre for Global Health, University of Split Medical School, Croatia

## Abstract

**Background:**

Meningococcal meningitis is a major cause of disease worldwide, with frequent epidemics particularly affecting an area of sub-Saharan Africa known as the “meningitis belt”. *Neisseria meningitidis* group A (MenA) is responsible for major epidemics in Africa.  Recently W-135 has emerged as an important pathogen. Currently, the strategy for control of such outbreaks is emergency use of meningococcal (MC) polysaccharide vaccines, but these have a limited ability to induce herd immunity and elicit an adequate immune response in infant and young children. In recent times initiatives have been taken to introduce meningococcal conjugate vaccine in these African countries. Currently there are two different types of MC conjugate vaccines at late stages of development covering serogroup A and W-135: a multivalent MC conjugate vaccine against serogroup A,C,Y and W-135; and a monovalent conjugate vaccine against serogroup A. We aimed to perform a structured assessment of these emerging meningococcal vaccines as a means of reducing global meningococal disease burden among children under 5 years of age.

**Methods:**

We used a modified CHNRI methodology for setting priorities in health research investments. This was done in two stages. In the first stage we systematically reviewed the literature related to emerging MC vaccines relevant to 12 criteria of interest. In Stage II, we conducted an expert opinion exercise by inviting 20 experts (leading basic scientists, international public health researchers, international policy makers and representatives of pharmaceutical companies). They answered questions from CHNRI framework and their “collective optimism” towards each criterion was documented on a scale from 0 to 100%.

**Results:**

For MenA conjugate vaccine the experts showed very high level of optimism (~ 90% or more) for 7 out of the 12 criteria. The experts felt that the likelihood of efficacy on meningitis was very high (~ 90%). Deliverability, acceptability to health workers, end users and the effect on equity were all seen as highly likely (~ 90%). In terms of the maximum potential impact on meningitis disease burden, the median potential effectiveness of the vaccines in reduction of overall meningitis mortality was estimated to be 20%; (interquartile range 20-40% and min. 8%, max 50 %). For the multivalent meningococcal vaccines the experts had similar optimism for most of the 12 CHNRI criteria with slightly lower optimism in answerability and low development cost criteria. The main concern was expressed over the cost of product, its affordability and cost of implementation.

**Conclusions:**

With increasing recognition of the burden of meningococcal meningitis, especially during epidemics in Africa, it is vitally important that strategies are taken to reduce the morbidity and mortality attributable to this disease. Improved MC vaccines are a promising investment that could substantially contribute to reduction of child meningitis mortality world-wide.

## Background

**Figure 1:**
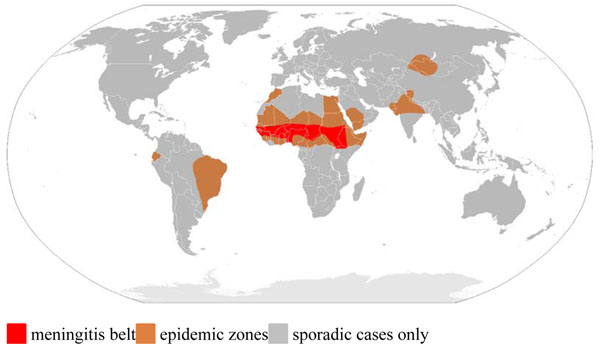
Demography of meningococcal meningitis (Source http://en.wikipedia.org/wiki/File:Meningitis-Epedemics-World-Map.png)

Meningococcal disease continues to be a major cause of childhood morbidity and mortality worldwide. The annual number of cases is conservatively estimated to be 1.2 million with at least 135,000 related deaths [1]. The majority of the deaths occur in developing countries. The area most significantly affected stretches across sub-Saharan Africa and has become known as the “meningitis belt” (Figure 1). Cyclic epidemics occur in this region every 5-12 years and exhibit a marked seasonality [2,3].

The incidence rate in epidemic years can reach 1000 per 100,000 population [4]. It has been estimated that since 1988 there have been over one million cases of meningitis in Africa [5]. The largest epidemic occurred in 1996–1997 across Africa, causing over 250,000 cases and 25,000 deaths. In recent years the reported number of meningitis cases has been increasing, with 41,526 cases in 2006, 45,997 in 2007, and 88,199 cases in 2009. This may reflect a new epidemic wave in sub-Saharan Africa [5].

The main etiological agents for bacterial meningitis are *Haemophilus influenzae*, *Streptococcus pneumoniae*, and *Neisseria meningitidis*[*6*]. Recently, the incidence of *Haemophilus influenzae* meningitis has declined following the introduction of the *Haemophilus influenzae* type b (Hib) conjugate vaccine [7,8]. *Neisseria meningitidis* is now estimated to account for 60-65% of all cases [2]. There are approximately 1.2 million cases of invasive meningococcal disease leading to 135,000 related deaths annually, according to the WHO [9]. In non-epidemic situations, pneumococcus is probably as common across all age groups as *Neisseria meningitidis* and causes three to five times more mortality. During major *Neisseria meningitidis* epidemics the proportion due to *Neisseria meningitidis* (and particularly MnA) is probably closer to 90%. Six capsular groups (A,B,C, W-135, X and Y) are associated with all invasive disease, groups B and C causing most cases in industrialised countries. *Neisseria meningitidis* serogroup A (MenA) was found to be the most frequent identified pathogen during epidemics in Africa [6,9]. However, in the years 2000 and 2001, serogroup W135 caused a major outbreaks during the annual Hajj pilgrimage in Saudi Arabia [10,11] and was also subsequently isolated from 80% of cerebrospinal fluid (CSF) samples in the 2002 epidemic in Burkina Faso and was also noted in other African countries [12]. Recently, groups X (mainly in Africa) and group Y (in the United States and other countries) have also emerged as important disease-causing isolates [9].

Different strategies have been implemented to stem the effects of the epidemics and their associated disease burden. Currently an emergency strategy of mass vaccination with MC polysaccharide vaccine following early detection of cases is instituted in the epidemic area [3]. However, this is a short-term solution and the strategy relies on timely surveillance and rapid response, both of which can be difficult to achieve in less-developed countries. Delays in vaccination can be associated with increased morbidity and mortality [13]. The polysaccharide vaccine against serogroup A does not prevent acquisition of nasopharyngeal carriage or confer herd immunity [14] and the vaccine response with repeated administration over time (for example with immunizations of at-risk populations during repeated epidemics) is uncertain [15]. The immunogenic response to the vaccine is diminished in infant and young children under the age of two years [16,17]. This is an important limitation given the epidemiology of meningococcal disease. These shortcomings may be overcome by the development of MC conjugate vaccines (named after the linking of the MC polysaccharide antigen to immunogenic carrier proteins) if optimally realized. However, some of these limitations are also true for the MC conjugate vaccines. For example the immunogenicity of a quadrivalent (A, C, W135, and Y) vaccine conjugated to diphtheria toxoid in infants is poor [9].

We aimed to assess the potential of the MC conjugate vaccines to reduce global under-5 meningitis mortality through the use of the CHNRI priority setting methodology.

## Methods

We used a modified Child Health and Nutrition Research Initiative (CHNRI) methodology for setting priorities in health research investments. The methodology has been described in great detail [18-22] and implemented in a variety of settings [23-28].

### CHNRI exercise – stage I: Identification and selection of studies

**Figure 2:**
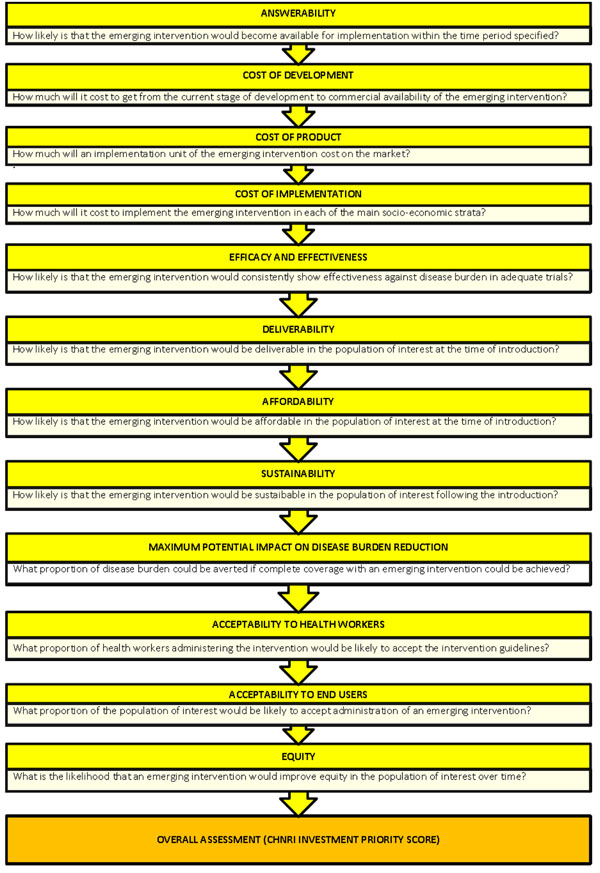
A summary of Stage I of the CHNRI process of an evaluation of emerging intervention (a systematic review of the key CHNRI criteria)

We applied the CHNRI method to estimate their potential impact of the emerging MC conjugate vaccines against two major serogroups of *Neisseria meningitidis* (A and W-135). We conducted a systematic literature review using the following 12 criteria: answerability, cost of development, cost of product, cost of implementation, efficacy and effectiveness, deliverability, affordability, sustainability, maximum potential impact on disease burden reduction, acceptance to health workers, acceptance to end users and equity (Figure 2). Details about the search strategies are presented in Additional file 1. The search was limited to Ovid MEDLINE (1999 to July 2009), EMBASE (1999 to July 2009), Web of Science (1999 to July 2009), Cochrane central register for controlled trials and a grey literature database (SIGLE). In order to ensure completeness, we also conducted hand searching of online journals, scanned the reference list of identified citations, and perused literature available on the websites of pharmaceutical companies and international agencies (GAVI and WHO).

Eligible studies were selected according to the pre-determined inclusion criteria. In particular, included studies investigated the answerability, effectiveness, deliverability, disease burden reduction or equity of vaccines or immunisation programs related to meningococcal conjugate or combination vaccines. Studies not eligible for inclusion were studies: (i) non-English language studies and (ii) developed world vaccine trials where developing world results for the same vaccine exist.

### CHNRI exercise – stage II: An expert opinion exercise

**Figure 3:**
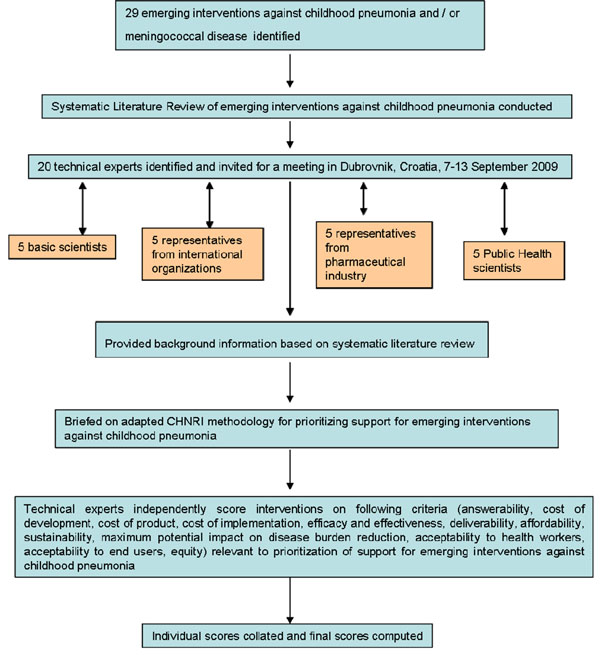
A summary of Stage II of the CHNRI process of an evaluation of emerging intervention (an expert opinion exercise using the CHNRI criteria)

We shared the initial review of the literature with 20 experts. The list of chosen experts included five leading basic scientists, five international public health researchers, five international policy makers and five representatives of the pharmaceutical companies. The 20 experts were chosen based on their excellent track record in childhood disease research, or prominent leadership roles in their organization related to this subject. We initially offered participation to the 20 experts with the greatest impact of publications in their area of expertise over the past 5 years (for basic researchers and international public health researchers), or for being affiliated to the largest pharmaceutical company in terms of vaccination programme or international agency in terms of their annual budget. For those who declined or who could adjust their schedules to participate (4 experts or 20%) replacements were found using the same criteria. The policy makers and industry representatives accepted our invitation on the condition of anonymity, due to sensitive nature of their involvement in such exercises. The experts met during September 7-13, 2009 in Dubrovnik, Croatia, to conduct the 2^nd^ stage of CHNRI expert opinion exercise. The process of second-stage CHNRI is shown in Figure 3. All invited experts discussed the evidence provided in CHNRI stage I, and then answered questions from CHNRI framework see Additional file 2. Their answers could have been “Yes” (1 point), “No” (0 points), “Neither Yes nor No” (0.5 points) or “Don’t know” (blank). Their “collective optimism” towards each criterion was documented on a scale from 0 to 100%. The interpretation of this metric for each criterion is simple: it is calculated as the number of points that each evaluated emerging intervention received from 20 experts (based on their responses to questions from CHNRI framework), divided by the maximum possible number of points (i.e., if all the answers from all the experts to all the questions were “Yes”).

## Results

After initial screening, sixty studies were finally included in the review. Thirty one of them were considered for assessing the answerability and effectiveness of monovalent and quadrivalent meningococcal vaccines covering MC serogroup A and W-135 and fourteen studies were considered to assess the deliverability, equity and reduction in global burden of disease from meningitis.

### Answerability

The conjugate vaccine boosts immunogenicity by transforming the vaccine from T-cell independent to T-cell dependant, thus allowing for priming of immunological memory and increasing immunogenicity in infants [3]. Conjugate vaccine technology has made significant advances in the past two decades [15] Hib conjugate vaccine was first licensed in 1987 followed by MenC conjugate in 1999 and pneumococcal conjugate (PCV7) vaccine in 2000 [29]. These conjugate vaccines were found to be safe and immunogenic in infants. The efficacy of these conjugate vaccines has demonstrated that the principle that the immunogenicity of bacterial cell surface polysaccharides can be improved by conjugating it with a protein carrier. Thus the development of the emerging MC conjugate vaccines is building on an established technology which has been shown to be successful.

The currently available MenC conjugate vaccines were first introduced in the UK in November 1999. Two different conjugated MenC conjugate vaccines were developed using CRM197 and one with tetanus toxoid as the carrier proteins [2]. These vaccines were licensed on the basis of immunogenicity and safety data but without a formal efficacy study. Studies reviewing the impact of MenC conjugate vaccines reported short term efficacy of 97% for teenagers and 92% for toddlers in England [30]. One year following introduction of these vaccines a 66% decrease in the prevalence of nasopharyngeal carriage of serogroup C meningococcal in adolescents [31] and 67% reduction in the attack rate in the unvaccinated adolescent population conferring a high level of herd immunity [32]. This experience therefore suggests that the development of further MC conjugate vaccines against other serogroups should, in principle, be a realistic possibility and could provide better protection against meningitis.

**Figure 4:**
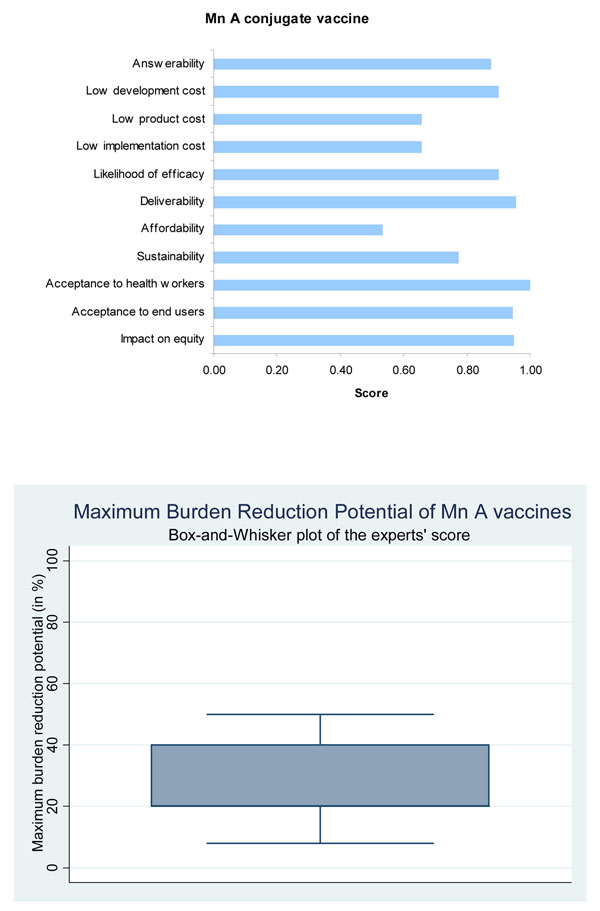
The results of Stage II CHNRI process – an expert opinion exercise assessing the potential usefulness of investment in Meningitis A conjugate vaccines

**Figure 5:**
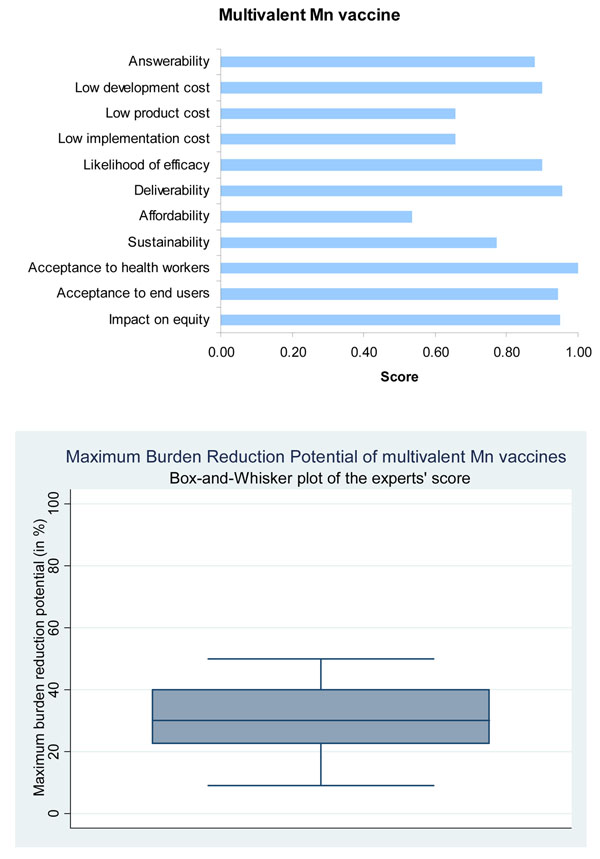
The results of Stage II CHNRI process – an expert opinion exercise assessing the potential usefulness of investment in multivalent meningococcal vaccines

The panel was very optimistic (score over 85%) about the ability of a monovalent conjugate vaccine against MenA to satisfy the criteria of answerability (Figure 4) while they were somewhat less optimistic (score about 75%) in the case of multivalent MC conjugate vaccines (Figure 5).

### Efficacy and effectiveness

#### Monovalent Conjugate Vaccine against Serogroup A

**Figure 6:**
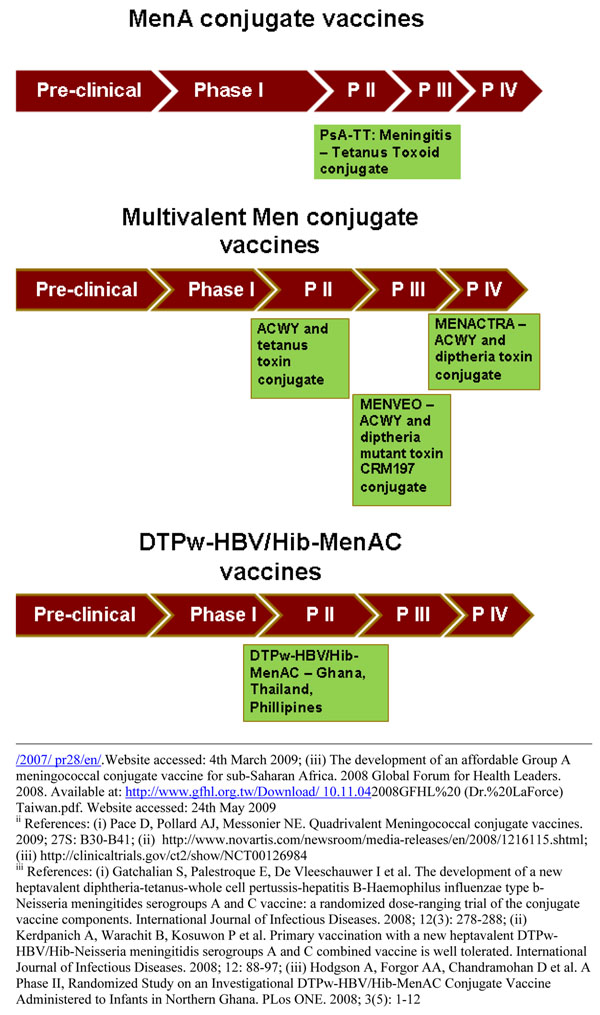
The current status of the research into MenA conjugate vaccines, multivalent Men conjugate vaccines and DTPw-HBV/Hib-MenAC vaccines as of September 2009 (see **Additional file**3 for details about the clinical trials phases; The DTPw-HBV/Hib-MenAC has now been discontinued).

A serogroup A meningococcal conjugate vaccine using tetanus toxoid as a carrier protein (PsA-TT conjugate vaccine) (MenAfrivax) has been developed at the Serum Institute of India Ltd. (SIIL) using a new licensed conjugation technique from the Center for Biologics Evaluation and Research/Food and Drug Administration (CBER/FDA, MD, USA) [33]. The vaccine demonstrated higher serum bactericidal antibody (SBA) than the PS-only vaccine in animal studies [34]. The first Phase I clinical trial was carried out in India among 18-35 year old healthy volunteers and results of the study showed PsA-TT to be safe, immunogenic and able to demonstrate long term functional antibody titers in adults (Figure 6) [35].

A Phase II clinical trial in Mali and Gambia evaluate the immunogenicity and safety of a single injection of PsA-TT vaccine in young children compared to a licensed meningococcal ACWY polysaccharide vaccine (PsACWY) and a licensed Hib conjugate vaccine (Figure 6**)**. The preliminary results showed 96% of the subjects in PsA-TT group had a four-fold increase in rSBA titer from week 0 to 4 as compared to 64% in the ACWY PS group and 36% in Hib group [36]. A Phase II/III clinical trial with 909 subjects of 2-29 year olds was carried out in Mali, Senegal, and the Gambia comparing the immunogenicity and long-term persistence of antibodies of a single dose of the PsA-TT vaccine with that of PsACWY. The preliminary findings were presented at International Pathogenic *Neisseria* Conference in 2008 which concluded that the PsA-TT vaccine is safe and consistently induced higher immune responses with respect to the licensed tetravalent polysaccharide vaccine. The immune response as measured by the MenA IgG ELISA concentrations ≥ 2 μg/mL was 100% (CI 99-100) in PsA-TT group and 88% (CI84-92) in the PsACWY group [37,38]. Preliminary results of similar Phase 2/3 study in India in healthy children of 2–10 years of age showed that the vaccine was safe and highly immunogenic [39]. Two Phase 3 clinical trials are currently being carried out in India and Mali to evaluate the safety and immunogenicity of a single dose of the PsA-TT vaccine [40].

Based on these, the experts were very optimistic (score over 85%) regarding the likelihood of efficacy of the monovalent conjugate vaccine against meningitis (Figure 4).

#### Multivalent Vaccines against Serogroup A and W-135

The two currently available meningococcal conjugate vaccines in developed world that target both MenA and MenW-135 along with serogroup C and Y are Menactra and Menveo.

Menactra (Meningococcal ACWY-diphtheria vaccine; MenACYW-D; Sanofi Pasteur) is a quadrivalent meningococcal protein–polysaccharide conjugate vaccine licensed for use in the United States for routine vaccination of 11–12 years old and for increased risk of invasive meningococcal disease (IMD) of 2–55 years old for both Canada and USA.

Menveo is a quadrivalent glycoconjugate vaccine, (MenACWY-CRM, Novartis Vaccines), formulated from oligosaccharides of MenA (10 g) and of Men C, W-135 and Y (5 g of each) and covalently linked to the diphtheria mutant toxin carrier protein, CRM197. In early 2010, the vaccine received approval from the Federal Drug Administration and European Medicines Agency for use among teenagers and adults.

These vaccines offer protection against four of the five most common meningococcal serogroups: A, C, Y and W-135.

**Figure 7:**
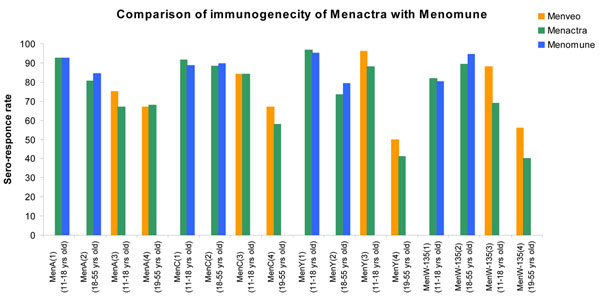
Seroresponse rate in Menactra and Menomune for MenA, MenC, MenY and MenW-135 ((1) Keyserling, H., et al: Safety, immunogenicity, and immune memory of a novel meningococcal (groups A, C, Y, and W-135) polysaccharide diphtheria toxoid conjugate vaccine (MCV-4) in healthy adolescents. *Arch Pediatr Adolesc Med*, 2005, 159: 907-13; (2) Bilukha, O.O. and N. Rosenstein*:* Prevention and control of meningococcal disease. Recommendations of the Advisory Committee on Immunization Practices (ACIP)*.* MMWR Recomm Rep, 2005, 54: 1-21; (3) Jackson, L. A., et al: Phase III comparison of an investigational quadrivalent meningococcal conjugate vaccine with the licensed meningococcal ACWY conjugate vaccine in adolescents. *Clin Infect Dis* 2009, 49: e1-10; (4) Reisinger, K. S., et al : Quadrivalent meningococcal vaccination of adults: phase III comparison of an investigational conjugate vaccine, MenACWY-CRM, with the licensed vaccine, Menactra. *Clin Vaccine Immunol* 2009, 16: 1810-1815.)

A randomized controlled trial was conducted among 11-18 years old comparing the immunogenecity of Menactra with Menomune (PSV-4; A/C/Y/W-135; Sanofi Pasteur Inc), a licensed tetravalent polysaccharide vaccine (Figure 6). The findings of the study showed a high percentage of subjects with fourfold or greater rise in serum bactericidal activity for all four antigens (the seropositivity was measured by Serum Bactericidal Assay with baby rabbit complement (rSBA)). The respective seroresponse rate in Menactra and Menomune for MenA, MenC, MenY and MenW-135 are presented in Figure 7[41]. Another randomized controlled trial conducted among persons aged 18–55 years compared immunogenecity of Menactra and that of Menomune at 28 days after vaccination showed similar results (Figure 7) [42]. A study in children aged 2–10 also found significantly higher seroresponse as measured by SBA activity in the Menactra group [43].

A similar phase III randomized controlled trial was carried out to compare the safety and immunogenicity of Menveo with that of Menactra. The results of the trial were published separately for the 11-to 18-year old and 19- to 55- year old groups [44,45]. The sero-respnose rates of Menveo and Menactra as measured by hSBA titre>= 1:8 at 1 month following the last dose for both age groups are presented in Figure 7. To evaluate immunogenicity and reactogenicity of Menveo in healthy infants, a study was conducted in Canada and the UK. It concluded that Menveo is immunogenic and well tolerated in infancy and could provide broad protection against meningococcal disease in this age group [46]. A new Quadrivalent Meningococcal (A, C, Y and W-135) Tetanus Protein Conjugate Vaccine (TetraMen-T) is in late stages of development from Sanofi Aventis (http://clinicaltrials.gov/ct2/show/NCT01049035) and a quadrivalent Meningococcal (A, C, Y, W-TT) Protein Conjugate Vaccine from GSK nearing licensure [47-49].

A novel heptavalent vaccine targeting *Neisseria meningitidis* serogroups A and C along with diphtheria, tetanus, whole cell pertussis-hepatitis B virus and Hib (DTPw-HBV/Hib-MenAC) was developed and was found to be safe and efficacious at generating immunological memory, particularly in infants in initial Phase I/II studies (Figure 6). A dose-ranging trial in the Philippines in 524 healthy infants showed that the DTPw-HBV/Hib-MenAC vaccine produced antibody responses comparable to the non-combination vaccines [50]. Another Phase II study on DTPw-HBV/Hib-MenAC in Ghana demonstrated adequate immunogenecity in infants [51]. However, currently there are no plans to take this vaccine forward for registration [52].

No randomized controlled clinical trial has been conducted to evaluate the effectiveness of these multivalent vaccines in developing world settings. Although immunogenicity studies usually can predict short-term effectiveness, the understanding of long-term protection needs well designed effectiveness trials. Moreover studies should be carried out to gather knowledge on link between immunogenicity and impact on nasopharyngeal carriage and herd immunity in the epidemic prone areas.

Based on the available information, the expert group was very optimistic (score over 85%) regarding the likelihood of efficacy of the multivalent conjugate MC vaccines against meningitis (Figure 5).

### Cost, affordability, deliverability and sustainability

The ongoing MenA clinical trials are currently evaluating the efficacy of different schedules of vaccination in infants. The Meningitis Vaccine Project (MVP) proposes delivery of the MenA conjugate vaccine in a two dose schedule, at 14 weeks and 9 months, or as a single dose at 9 months [2]. A catch-up from age 1 to 29 years is planned for each meningitis belt country. This permits incorporation of the vaccine into existing EPI schedules, since it will be given concurrently with the final DPT dose (DTP3) at 14 weeks and with the measles vaccine at 9 months [53]. This should act to promote high coverage of the MenA vaccine without the need to commit very substantial further resources for promotion and delivery of a new immunisation programme. In 2006, the average uptake of the DTP3 vaccine across the African region was 73%, with similar rates of coverage for the measles vaccine [54]. However, this masks pronounced disparities between countries, in the “meningitis belt”. For example, Niger achieved <40% coverage for DTP3 while rates in Burkina Faso and the Gambia were >90%, which suggests that approaches to vaccine delivery must be modified in areas where current strategies have low uptake. Such countries may benefit from the implementation of periodic regional campaigns to boost immunisation coverage [54].

Provision of viable vaccines relies on appropriate storage and transport and a functioning “cold-chain”. Currently, there is a significant wastage of vaccines in developing countries due to inadequate funding, poor equipment and lack of training of health-care workers [55]. Storage is an important factor to address as the MenA Ps, from which the conjugate is derived has been shown to be the least stable meningococcal Ps [56] and any deviation from the recommended 2-8°C storage temperature [55] could render the vaccine unusable. This is also applies to the multivalent conjugate vaccines.

The greatest barrier to uptake of vaccines by developing countries is cost [57]. The MVP was been granted $70 million by The Gates Foundation [57] and $29.5 million from The Global Alliance for Vaccines and Immunisation (GAVI) [58], which should allow Men A conjugate vaccine to be provided at $0.40 a dose, in agreement with the manufacturer, the Serum Institute of India Limited [3]. Currently the MVP aims to provide the Men A conjugate vaccine at a lower cost through push financing. MenAfriVac™ has received WHO prequalification (which guarantees that individual vaccines meet international standards of quality, safety, and efficacy). The producer, Serum Institute of India Ltd, has received marketing authorization for export and use of MenAfriVac™ in Africa and mass introduction will start in late 2010 in Mali, Burkina Faso and Niger with backing from Global Alliance for Vaccines and Immunization (GAVI) and WHO. Given the emergence of the importance of the W-135 and X serogroups in Africa, the program should be flexible enough to introduce multivalent conjugate vaccines targeting A, W-135 and X serotypes if necessary.

The panel was highly optimistic (score around 90%) that a monovalent conjugate vaccine could be developed at a low cost (Figure 4), while they were only moderately optimistic (score about 70%) in case of multivalent vaccines (Figure 5). They were however optimistic that once introduced with support from donor agencies like GAVI Alliance the vaccine delivery was sustainable. The main concern related to both vaccines was expressed over the cost of product, its affordability and cost of implementation (Figures 4 and 5).

### Burden of disease reduction

In 2009, 88,199 cases of suspected meningococcal disease were reported in “meningitis belt” countries, resulting in 5,352 deaths. The worst hit areas were Nigeria and Niger which combined accounted for 69,577 cases and 3,046 fatalities. From 2003-2007, 87.8% of epidemic cases were attributable to serogroup A and 10% to W135 [59]. Reactive mass vaccination of epidemic districts can prevent up to 70% of cases, if the vaccine administration happens on time [35]. If MenA conjugate vaccine can reproduce the effect of the MenC conjugate vaccine, which prompted a 90% decrease in cases and 70% reduction in rates of carriage in UK [6], then an even greater reduction in the number of cases can be expected in Africa. However, this will be determined by the ability to efficiently deliver the vaccine and obtain maximum coverage. The effect of a quadrivalent vaccine on disease reduction is dependant on the uptake of such a vaccine in the MVP program as well as on virulence/carriage of the organism, levels of antibodies needed to eliminate carriage, duration of protection, and other factors. There have been suggestions that the inclusion of other serogroups into vaccination programs may lead to further disease burden reduction in this region [58]. A recent review of meningococcal carriage during epidemics showed W135 and Y causing a greater number of cases [60]. Combination vaccines with non-EPI diseases may also be beneficial in providing countries with protection against both meningococcal disease and those not currently part of that country’s EPI schedule. This may lead to multi-disease burden reduction as demonstrated in Ethiopia but given the fact that further development of combination vaccine DTPw-HBV/Hib-MenAC has ceased, we may need to wait longer to get the benefit of such a vaccine [61].

The expert group felt that both the monovalent MC conjugate vaccines and the multivalent MC conjugate vaccines had high median potential effectiveness for reduction of meningitis mortality (20%; interquartile range 20-40% and min. 8%, max 50%; and 30%; interquartile range 25-40% and min. 9%, max 60%, respectively) (Figures 4 and 5).

### Acceptability and equity

A limitation to the improvement of equity is that it appears inevitable that a health-promotion strategy similar to those currently employed will reach higher-income populations prior to the underprivileged, unless a novel method of introduction is found [62]. This effect is particularly pronounced in low and middle income countries in Asia and Africa, where greater inequities exist [62]. As herd immunity is dependant on high levels of coverage [2], it is vital that immunizations are delivered to the poorest areas which are most affected by disease. The MVP financing strategy for MenA [3] may allow more periodic immunization campaigns, which were shown to be effective in Africa [54]. More money must be spent on strengthening delivery capacity in these countries to ensure equity is improved. However multivalent vaccine may cause further inequity due to the high price of that vaccine. Currently a trivalent A/C/W135 PS polysacharide vaccine costs US$ 1.30 per dose [3] which means a novel quadrivalent conjugate vaccine will be more expensive and be out of reach for the majority of the population of African countries. The decision to develop only a low cost Men A conjugate vaccine through push financing was based on the expectation that other serogroups especially W-135 will not cause major epidemics. However, in future the control program may still need to consider the development of a low cost multivalent vaccine too through push financing.

The expert group were very optimistic (score over 90%) that both the monovalent and multivalent MC conjugate vaccines would have a positive impact on equity, and would be acceptable to both health workers and end users (Figures 4 and 5).

## Discussion

The literature review summarized in this paper presents available evidence that is useful in considering the relative priority for investment in emerging MC conjugate vaccines. The scores of both monovalent MC conjugate and the multivalent MC conjugate vaccines are presented against a common set of criteria. These scores reflect the “collective optimism” of a panel of experts drawn from varying backgrounds. We have shown that both the monovalent MC conjugate and multivalent MC conjugate vaccines are considered to have the potential to significantly reduce the burden of meningococcal meningitis in children under the age of 5 years. Countries in the meningitis belt in general, and the poorer nations in particular, account for the greatest global burden of disease due to meningitis. An effective vaccine distributed worldwide will reduce that burden, and if delivery is targeted at the poorest areas, the inequity gap in health will also be reduced.

For MenA MC conjugate vaccine the experts showed a high level of optimism (~ 90%) for 7 out of the 12 criteria. The expert group felt that the likelihood of efficacy on meningitis was very high (~ 90%) and the maximum potential impact on disease burden was also high. Median potential effectiveness of the vaccines in reduction of overall meningitis mortality was predicted to be 20% (interquartile range 20-40% and min. 8%, max 50%). The MenA conjugate vaccine scored well on answerability, low development cost, likelihood of efficacy against meningitis, deliverability, acceptability to health workers and end users, effect on equity and maximum potential to reduce the burden of mortality due to meningitis. The multivalent vaccines scored similarly well on all criteria except answerability and low development cost. The main concern related to both vaccines was expressed over the cost of product, its affordability and cost of implementation

Both the monovalent and quadrivalent vaccines have been shown to have a good safety profile, with high immunogenicity against MenA and MenW-135 in young children and adults across Phase II/III trials. However it is important that well designed controlled studies are carried out in developing world settings to provide data on effectiveness. Following demonstration of the viability of the MenC conjugate vaccine stored at room temperature, a similar study examining the effect of storage temperature on the new conjugate vaccines would be invaluable. This would potentially allow reassessment of deliverability, particularly to communities with limited cold chain facilities.

The CHNRI methodology was primarily designed to evaluate existing interventions and competing investment priorities for health research. Though we used the CHNRI criteria, we modified it by including systematic review of available literature and not involving all stakeholders (e.g. end-users and health workers). The scores included herewith express the collective opinion of a panel of 20 experts. While there is always an element of uncertainty in predicting impact of interventions which do not exist and have no clinical trial data to support them, we feel that the results would be reproducible with another panel in a different setting. The literature review also had some limitations. Firstly, the literature search was limited to selected databases and to articles published in English. In addition, a variety of key-words yielded results, especially when searching the domains “deliverability” and “equity”. Although every effort was made to be inclusive, such a broad range increases the chance of missing information that may be important. Secondly, it was not always possible to find current literature specific to meningococcal vaccines. In these circumstances relevant data on other related vaccines was sought and this may not always be fully appropriate. Finally, due to marketing patents, retrieval of the most recent information on the progress of a vaccine was generally difficult.

## Conclusions

With increasing recognition of the burden of meningococcal meningitis, especially during epidemics in Africa, it is vitally important that steps are taken to reduce the morbidity and mortality attributable to this disease. The strengthening of the surveillance system is important to support any vaccination program. The increase in incidence of W135 in recent epidemics raises the question of whether it would be more appropriate to use a multivalent vaccine with A and W135 in the “Meningitis Belt” instead of a monovalent vaccine. New Initiatives are joining MVP to strengthen the international effort to eliminate meningococcal epidemics in Africa. A new international consortium (MenAfriCar) http://www.menafricar.org/ has started working in Africa and aims to study patterns of meningococcal carriage and transmission in this region, as well as documenting the impact of any new MC conjugate vaccine. MenAfriCar aims to further develop regional capacity for delivery of immunization programmes. However, success will rely on concerted and sustained commitment from governments, charities and health care workers to implement those vaccination strategies shown by research to be the most effective and practical/feasible.

## Competing interests

The authors declare that they have no competing interests.

## Authors' contributions

All authors of this research paper have directly participated in the planning, execution, or analysis of the study and have read and approved the submitted version. In particular IR and HC designed the study and directed its implementation, including quality assurance and control. DC and TH were responsible for the acquisition of the data and conducted the literature review. ET, HN, LZ, RF, IL, HLJ, SEA, CBN and RB helped design the study’s analytic strategy and prepared the Materials and Methods, Results and Discussion sections of the text. All authors of this research paper have critically revised the manuscript for important intellectual content.

## Supplementary Material

Additional file 1 Additional search terms Click here for file

Additional file 2 Questions used in the Phase II CHNRI process Click here for file

Additional file 3 The clinical trial process Click here for file
