# Supplementary material for: An evaluation of emerging vaccines for childhood meningococcal disease
Source: BMC Public Health. 2011 Apr 13;11(Suppl 3):S29. doi: 10.1186/1471-2458-11-S3-S29 (PMC3231902; doi:10.1186/1471-2458-11-S3-S29)
Supplement: Additional file 3 — The clinical trial process [file 1471-2458-11-S3-S29-S3.doc]

**Additional file 3 – The clinical trial process**

| Study | Purpose | Population under study | Study duration |
| --- | --- | --- | --- |
| Pre-licensure  studies |  |  |  |
| Phase I | To evaluate clinical tolerability and reactogenicity of vaccine | Small number of highly-selected normal healthy adult volunteers | Short duration |
| Phase II | To provide preliminary information on biological activity of elicited antibodies to predict vaccine efficacy using standardized serological assays  To further evaluate safety  To evaluate immunological activity, and dose-ranging and 'optimal' schedules of vaccination  To provide clinical evidence of consistency of vaccine manufacturing | Larger numbers of individuals who may more closely resemble  the ultimate target population | Medium duration |
| Phase III | To demonstrate safety, efficacy, and clinical protection of vaccine in a large population  To evaluate immunogenicity using standardized serological assays  To assess duration of protection | Large sample of individuals from the intended target population | Long duration for randomized  placebo-controlled  trials  Medium duration  for active-control  immunogenicity  trials |
| Post-licensure  studies |  |  |  |
| Phase IV | To evaluate impact of vaccine at population level, where effects of vaccination also depend on coverage, distribution of vaccine, and efficacy preventing disease and colonisation | Overall population under surveillance  Restricted sample of the population  required for vaccine-effectiveness case-control study | Medium duration |
